# 

**Published:** 1903-04-04

**Authors:** 


### IMPORTANT NOTICE.

**On account of the Easter Holidays, we beg to announce that we shall go to press with our issue of the 11th APRIL on TUESDAY NIGHT, the 7th APRIL. Advertisements intended for insertion in this issue should therefore reach us by 4 p.m. ON TUESDAY, the 7th APRIL.**

### NOTICE TO CORRESPONDENTS.

All MSS., Letters, Books for Review, and other matters intended for the Editor should be addressed The Editor, "The Hospital," The Hospital Buildings, 28 & 29 Southampton Street, Strand, W.C.

The Editor cannot undertake to return rejected MS., even when accompanied by stamped directed envelope.

**Notice to Advertisers and Subscribers.**

All Advertisements. Orders for Copies of The Hospital, and Business Communications should be addressed to **THE MANAGER, (Not to the Editor)**, The Hospital, 28 & 29 Southampton Street, Strand, London, W.C.

The Cost of Subscription, post free, is as follows.—Per week, 3½d.; per quarter, 3s. 9d.; per half-year, 7s. 6d.; per annum, 15s. Foreign and Colonial:—Per annum, 19s. 6d., payable, in all cases, in advance.

**NOTICE.—Vol. XXXIII. of "The Hospital" (October 1902 to March 1903) in handsome cloth binding, will shortly be on sale at the office of this Journal, price 7s. 6d. Cases for binding the Numbers contained in this Volume 1s. 6d. Index to Vol. XXXIII., 2½d., post free.**

**The monthly part for April is now ready. Price 6d., by post 8d.**

### BOOKS RECEIVED.

The Scientific Press, Ltd.

"Burdett's Official Nursing Directory, 1903."

"Uniform System of Accounts for Hospitals, Charities, Missions, and Public Institutions." By Sir Henry Burdett, K.C.B.

The Walter Scott Publishing Co., Ltd.

"Indigestion: its Prevention and Cure." By F. H. Alderson.

J. Wright and Co., Bristol.

"The Medical Annual, 1903."

"First Aid to the Injured and Sick." By Warwick and Tunstall.

Hodder and Stoughton.

"Famishing London." By F. A. McKenzie.

H. K. Lewis.

"Disinfectants and Antiseptics." By E. T. Wilson.

Debenham and Freebody.

"A Dress Review." By Mrs. Aria.

### The Student of Medicine.

### APPOINTMENTS.

**W. B. Barclay,** M.D., D.P.H.Vict., appointed Medical Officer of Health to the Farnborough Town Council. **Mildred M. Burgess,** M.B.Lond., appointed Junior House Surgeon to the Victoria Hospital for Sick Children, Hull. **C. M. Chadwick,** M.D.Oxon., F.R.C.P.Lond., appointed Honorary Consulting Physician to the Leeds Public Dispensary. **J. F. Hall Dally,** M.R.C.S.Eng., L.R.C.P.Lond., appointed Assistant Resident Medical Officer to the Royal National Hospital for Consumption and Diseases of the Chest, Ventnor. **E. L. Dewdney,** L.S.A., appointed District Medical Officer of the Newton Abbot Union. **Margaret B. Dobson,** M.B.Lond., appointed Senior House Surgeon to the Victoria Hospital for Sick Children, Hull. **Herbert Filton,** L.R.C.P.Edin., M.R.C.S.Eng., appointed Surgeon to the Dewsbury and District General Infirmary. **John Fullard,** M.R.C.S.Eng., L.S.A., appointed District Medical Officer of the Hexham Union. **C. P. Lapage,** M.B., Ch.B.Vict., appointed Junior Resident Medical Officer to the Manchester Children's Hospital, Pendlebury. **Philip Richard Littleton,** M.R.C.S.Eng., reappointed Medical Officer of Health to the Ashbourne Urban District Council. **Edward Malins,** M.D., F.R.C.P., appointed Honorary Consulting Obstetric Officer to the General Hospital, Birmingham, after 25 years' service. **Antony Alexander Martin,** M.D.Lond., B.S., M.R.C.S.Eng., L.R.C.P.Lond., D.P.H.Lond., appointed Medical Officer to the Eastbourne and Seaside District of the Eastbourne Union. **E. J. F. Moore,** M.R.C.S., L.R.C.P.Lond., appointed Medical Officer of the Well Street Workhouse of the Poplar Union. **Herbert Newsome,** M.B., B.S.Durh., appointed District Medical Officer of the Long Ashton Union. **Frederick L. Morris,** M.B., C.M.Glasg., appointed Chief Government Medical Officer of Antigua. **A. W. Swettenham,** L.R.C.P., L.R.C.S.Edin., appointed District Medical Officer of the Leominster Union. **Walter H. Maxwell Telling,** M.D., B.S.Lond., M.R.C.P., appointed Honorary Physician to the Leeds Public Dispensary. **C. L. Traylen,** L.R.C.P.Lond., M.R.C.S.Eng., appointed House Physician to the Royal Hospital for Diseases of the Chest, City Road, E.C. **H. E. Watkins,** M.R.C.S., L.R.C.P.Lond., appointed Certifying Factory Surgeon for the Newton-le-Willows District of the county of Lancaster.

### "The Hospital" Institutional Library.

"Hospitals and Asylums of the World." 4 Vols., with a Portfolio of Plans, complete £12 12s.

"Burdett's Hospitals and Charities, 1902." 5s.

"Burdett's Official Nursing Directory, 1903." 3s. net.

"Cottage Hospitals, General, Fever, and Convalescent." 10s. 6d.

"Uniform System of Accounts for Hospitals and Public Institutions." New Edition. 4s. net.

"Hospital Expenditure: The Commissariat." 2s. 6d.

"A Legal Handbook for the Use of Hospital Authorities." 2s. 6d.

"The Cottage Hospital Case Book or Register of Patients." 10s. and 12s. 6d.

"The Furnishing and Appliances of a Cottage." 6d.

All these are published by the Scientific Press, Ltd., and may be obtained through any bookseller, or direct from the publishers, 28 and 29 Southampton Street, Strand, London, W.C.

